# Prediction of muscle activity during loaded movements of the upper limb

**DOI:** 10.1186/1743-0003-12-6

**Published:** 2015-01-15

**Authors:** Robert Tibold, Andrew J Fuglevand

**Affiliations:** Departments of Physiology and Neuroscience, University of Arizona, PO Box 210093, Tucson, AZ 85721-0093 USA

**Keywords:** Functional electrical stimulation, Electromyography, Artificial neural networks, Kinematics, Grip force, Upper limb

## Abstract

**Background:**

Accurate prediction of electromyographic (EMG) signals associated with a variety of motor behaviors could, in theory, serve as activity templates needed to evoke movements in paralyzed individuals using functional electrical stimulation. Such predictions should encompass complex multi-joint movements and include interactions with objects in the environment.

**Methods:**

Here we tested the ability of different artificial neural networks (ANNs) to predict EMG activities of 12 arm muscles while human subjects made free movements of the arm or grasped and moved objects of different weights and dimensions. Inputs to the trained ANNs included hand position, hand orientation, and thumb grip force.

**Results:**

The ability of ANNs to predict EMG was equally as good for tasks involving interactions with external loads as for unloaded movements. The ANN that yielded the best predictions was a feed-forward network consisting of a single hidden layer of 30 neural elements. For this network, the average coefficient of determination (R^2^ value) between predicted and actual EMG signals across all nine subjects and 12 muscles during movements that involved episodes of moving objects was 0.43.

**Conclusion:**

This reasonable accuracy suggests that ANNs could be used to provide an initial estimate of the complex patterns of muscle stimulation needed to produce a wide array of movements, including those involving object interaction, in paralyzed individuals.

## Background

Over the past few decades, functional electrical stimulation (FES), involving artificial activation of skeletal muscles, has been used to partially restore limb function in paralyzed individuals [1–16]. However, only a few pre-programmed movements are permitted by most existing systems. This limitation is related to two major challenges. One (and the topic addressed in the present study) relates to the difficulty associated with identifying the intricate patterns of muscle activity needed to produce even relatively simple movements. Most natural movements require coordination of many muscles across multiple joints and such complex systems do not readily lend themselves to analytical solutions. And two, even if a flexible system were developed that could deliver appropriate patterns of muscle stimulation associated with a wide array of movements, the challenge remains as to how a paralyzed individual would provide the command signal needed to specify the desired behavior.

To address the first of these challenges, we have used machine-learning methods, including probability-based algorithms and artificial neural networks, to predict activity patterns across several muscles associated with complex movements of the arm and hand [17–19]. In addition, we have shown that such machine-learning algorithms trained using data from one individual can be used to accurately predict patterns of muscle activity in other individuals. This implies that an FES system using this strategy could be trained on able-bodied subjects and then be deployed, at least as a first approximation, in paralyzed individuals. Furthermore, we have shown that such predicted patterns of muscle activity can be transformed into trains of stimulus pulses that elicit desired motor behaviors with reasonable fidelity [17, 20].

A shortcoming of our previous work, however, is that we restricted predictions of muscle activity to free movements of the arm and hand not involving external contact forces. Clearly, the utility of a system for controlling FES necessitates that it predicts patterns of muscle stimulation needed for manipulation of objects in the environment. In theory, it should be possible to include digit contact forces detected with artificial sensors as an additional set of inputs, along with limb kinematics, to predict patterns of muscle activity associated with a wide range of motor behaviors including those involving interaction with objects. Here we tested this idea by predicting electromyographic (EMG) signals using artificial neural networks in 12 upper limb muscles while human subjects grasped and moved objects of different weights and dimensions.

## Methods

### Experimental setup and EMG recording

Experiments were performed on nine healthy adult subjects (age: 21–31, gender: 2 females, 7 males). All subjects signed informed consent approved by the institutional human subjects committee (University of Arizona, Human Subjects Protection Program, Internal Review Board). The procedures used here were similar to those we have used previously [18, 19]. In the present study, we predicted muscle activity associated with transporting the hand throughout peri-personal space with or without an external load applied to the limb. We did not, however, attempt to predict hand muscle activity associated with gripping and manipulating objects.

**Figure 1:**
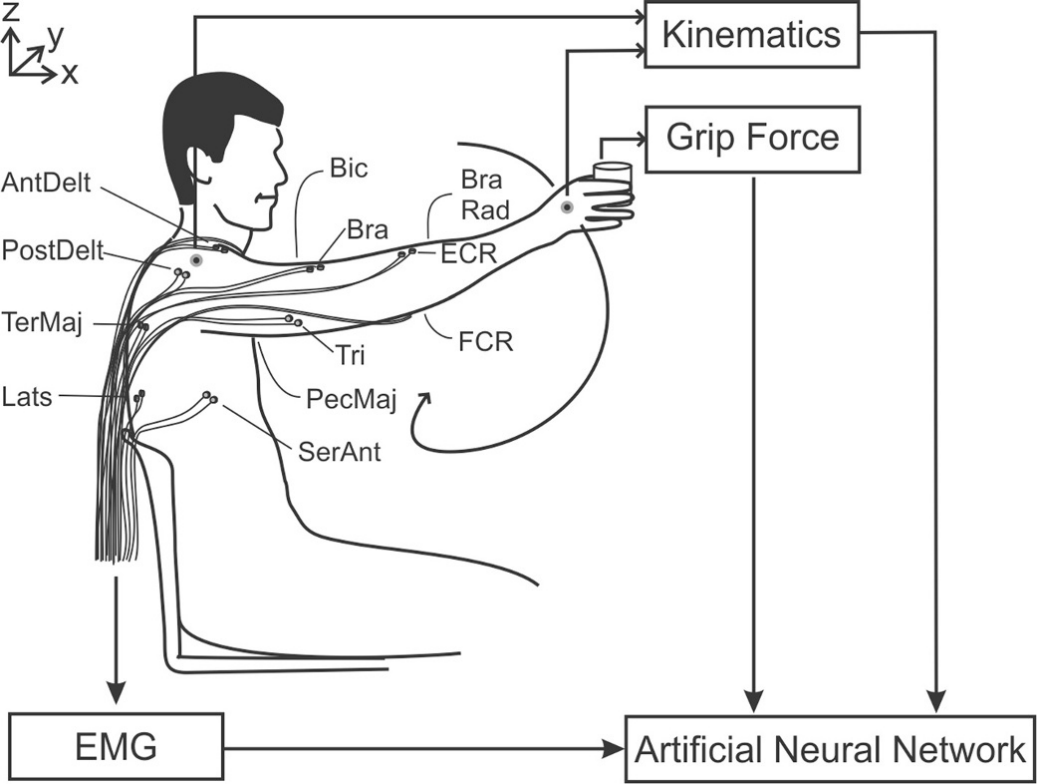
**Experimental setup.** Surface EMG signals from 12 arm muscles, kinematic data from electromagnetic sensors placed on the hand and shoulder, and grip force exerted by the thumb were recorded while healthy individuals executed random, unrestricted movements without (unloaded) or with (loaded) objects in their hand. These data served as inputs to train artificial neural networks. Once trained, a new set of kinematic and grip force data served as inputs to the artificial neural network in order to predict the associated patterns of EMG activity across the 12 muscles (Lats: latissimus dorsi, PecMaj: pectoralis major, Teres: teres major, SerAnt: serratus anterior, AntDelt: anterior deltoid, PostDelt: posterior deltoid, Tricep: triceps brachii, Bicep: biceps brachii, Brach: brachialis, BrachRad: brachioradialis, ECR: extensor carpi radialis, FCR: flexor carpi radialis) (adapted from Johnson and Fuglevand, 2009).

Subjects sat upright on a low-back, wooden chair without armrests (Figure 1). EMG signals were recorded using surface electrodes from 12 muscles of the right arm (serratus anterior, anterior deltoid, posterior deltoid, pectoralis major, latissimus dorsi, teres major, biceps brachii, brachialis, brachioradialis, triceps brachii, extensor carpi radialis and flexor carpi radialis). Target locations for placing EMG electrodes were based on surface anatomy and palpation with reference to an EMG atlas [21]. One exception was the brachialis for which electrodes were placed on the lateral surface of the upper arm midway between the distal insertion of the deltoid and the proximal insertion of the brachioradialis.

For each muscle, conductivity gel was applied inside the ceramic housing of Ag-AgCl electrodes that were then fixed on the skin in a bipolar configuration (~2 cm interelectrode spacing) using adhesive discs and surgical tape. Electrode cables were secured along the length of the limb using elastic wrap (3 M Vetwrap) to minimize cable movement artifacts. EMG signals were differentially amplified with a gain of 1000 and band-pass filtered between 100 and 475 Hz (Lynx-8 amplifiers, Neuralynx, Bozeman, MT). EMG signals were digitally sampled at 2500 Hz/channel by the data acquisition system (CED Power 1401, Cambridge, UK). We used a relatively aggressive high-pass filter (100 Hz) to minimize cable movement artifact and because such high-pass filtering appears to provides a better representation of the underlying active state of muscle than conventional filtering and also yields better predictions of mechanical variables (such as muscle force) than conventional filtering [22].

### Grip force detection

Humans interact with objects in the environment predominantly with the hands. Contact, load, and inertial forces associated with such object interactions can set up significant torques across multiple joints of the upper limb that require complementary adjustments in muscle activity to effect controlled manipulation. Previous studies have shown that the grip forces applied normal to an object are proportional to the load [23, 24] and that grip forces are precisely modulated to account for additional inertial forces when objects are moved by the upper limb [25, 26]. Furthermore, for many types of grips, the normal force exerted by the thumb counterbalances the forces exerted by the other digits in contact with the object [2, 27, 28]. As such, the normal forces exerted by the thumb provide an indirect indication of the external forces encountered by the hand during interactions with objects.

Accordingly, we monitored the normal forces on the distal segment of the thumb using a flexible force-sensing resistor (FSR 402, Interlink Electronics, Camarillo, CA). The circular sensor (1.3 cm diameter, 0.2 N actuation force, accuracy ± 2%) was fixed to a thin, adjustable leather glove (TaylorMade Tour golf glove) with double-sided adhesive. Different sized gloves were used to accommodate hands of different sizes. Once the glove was donned, a thin layer of elastic wrap was used to encircle the distal segment of the thumb to cover the sensor with a material having a relatively high coefficient of friction. The leads from the sensor were taped to the glove and connected to a custom-built amplifier via a long lightweight cable. The thumb contact force signal was also sampled by the data acquisition system at 2500 Hz.

### Kinematics

Electromagnetic tracking (Liberty System, Polhemus Inc., Colchester, VT, USA) was used to record (120 Hz/channel) six degrees-of-freedom (x, y, z positions and roll, pitch, and yaw orientations) motion of the hand and shoulder (Figure 1). One position sensor was taped to the shoulder just lateral to the acromion process and a second sensor was fixed to the dorsum of the hand using elastic wrap. These sensors detect the magnetic field strength emitted by an electromagnetic source coil that was fixed to the back of the chair. The sensors have a resolution of 0.025 mm (at 1 m from source coil) and a static accuracy of 0.76 mm RMS error. Synchronization pulses generated by the Polhemus system at 120 Hz and timed to each data acquisition cycle were recorded by the CED data acquisition system to facilitate off-line alignment of kinematic data to the recorded EMG and contact force signals.

### Experimental procedures

Once EMG electrodes and sensors were in place, subjects performed a series of maximum voluntary contractions (MVCs), one for each muscle recorded. Each MVC involved an ~ 2 s isometric contraction resisted by co-contraction of other muscles and by bracing action of the contralateral limb. Subjects were instructed as to the maneuver best thought to optimally activate the target muscle. Subjects performed two MVCs for each muscle.

Subjects were then asked to perform unrestricted random movements of the arm without an object in their hand during which EMG and kinematic data were recorded. Subjects were instructed to move using a wide range of velocities, to trace out a variety of trajectories, and to encompass the entire reach space while keeping their trunk against the back of the chair. Subjects were encouraged to rest if needed (with the arm hanging pendant at the side). The entire duration of this procedure was ~ 25 min. For simplicity, we refer to these movements as *unloaded*.

Following an ~ 10 min rest period, subjects were again asked to perform unrestricted random movements during which they occasionally picked up and moved continuously one of four brass cylinders of differing weights (100, 200, 500, and 1000 g) and dimensions (2.3 × 3.3, 3.0 × 3.8, 4.0 × 6.0, and 5.0 × 8.0 cm, diameter × height, respectively). Grasping involved object contact with the distal segments of the digits, usually the index and middle fingers, opposed by the thumb. Again, subjects were encouraged to move using a wide range of velocities and trajectories including changing the orientation of the cylinder using wrist supination/pronation, flexion/extension, and radial/ulnar deviation. Each ~ 2 minute period of moving a load was followed by an ~ 2 min movement period without a load. Loads were picked up in random order from a small platform positioned lateral to the thigh at knee height. Each load was handled in this way twice and subjects again were encouraged to rest if needed. The total duration of this procedure was about 32 minutes. We will refer to these movements as *loaded* (keeping in mind that *zero* load was also sampled). Thumb contact force, EMG, and kinematics were sampled continuously during these procedures. No subjects reported muscle fatigue.

### Kinematic data processing

All data were processed offline in Matlab (Mathworks, Natick, MA). Hand position (x - anterior/posterior; y - medial/lateral; z - vertical) data were expressed relative to the shoulder position and normalized to the maximal displacement of the hand recorded over the entire session. Pitch, roll, and yaw orientations of the hand were expressed relative to an earth-based reference frame. These data were then low-pass filtered (6 Hz cut-off, sixth order Butterworth, zero phase).

### EMG data processing

Any modest DC offset was first removed from EMG signals using a high-pass filter (cutoff 0.1 Hz, sixth order Butterworth). EMG signals were then full-wave rectified and low-pass filtered at 2 Hz (sixth order, Butterworth, zero phase). EMG signals were then down-sampled and synchronized to the kinematic data (120 Hz). Muscle activation values were then normalized to the maximum amplitude detected for each muscle during the MVC trials.

### Artificial neural network structures

Previously, we demonstrated the applicability of using artificial neural networks (ANN) for predicting EMG patterns from kinematics in the absence of external forces [19]. Here we used a similar ANN architecture but included thumb contact force as an input in addition to the kinematic parameters. Six kinematic parameters were used as inputs (x, y, z position of the hand relative to the shoulder, and pitch, roll, and yaw orientations of the hand). Additional kinematic parameters were not included, as we previously have shown that inclusion of other features (such as additional limb landmarks, velocities, accelerations) had only modest effects on predictions of EMG patterns [18].

The ANN was a multilayer perceptron (MLP) involving a feed-forward network created in the Neural Networks Toolbox of Matlab. It contained four hidden layers. The first hidden layer had 20 neurons, the second and third layers each had 9 neurons, and the fourth layer had 20. The network was built with two time delays - the kinematic and force values from the two previous time steps were also included as inputs. The network was fully connected so that in every layer all of the individual neurons received all of the outputs from neuronal units involved in the previous layer. A hyperbolic-tangent sigmoid function was used as the transfer function for the neuronal units. In the output layer, the 20 neurons of the fourth hidden layer were fully connected to the 12 muscle outputs (predicted EMG signals) using a linear transfer function. Network initialization was done with random weights and biases. Training was repeated for 100 iterations using gradient descent with momentum weight and bias learning function, the backpropagation training function, and a mean-squared error performance function.

We also tested other ANNs to determine whether simpler (and computationally more efficient) architectures could yield equivalent predictions. We used the same multilayer perceptron (MLP) structure as in the nominal (and more complex) network but with only a single hidden layer comprised of different numbers of processing units (1, 2, 5, 10, and 30). We refer to these networks as MLP1, MLP2, MLP5, MLP10, and MLP30, and the complex network as MLP20_9_9_20.

### Data analysis

Kinematic and EMG data, along with grip force exerted by the thumb, were recorded from 9 subjects during the execution of complex arm movements without (*unloaded* movements) or with (*loaded* movements) loads in their hand. For simplicity, most of the data analyses were carried out for within-subject predictions of EMG. Namely, the data used to train neural networks and the data used for testing originated from the same subject. We have previously shown effective prediction of EMG signals across subjects (i.e. training data and test data obtained from different subjects) using similar machine-learning approaches [17–19].

For both unloaded and loaded movements, the initial 1000 s (~17 min) of data was selected to serve as the training set for each subject. For the *loaded*-movement task, this time period encompassed one trial with each of the four external loads in addition to intervening episodes with zero load. The remaining ~ 8 min of data from the *unloaded* movements and ~ 15 min of data for the *loaded* movements were designated as the test sets. The duration of the *loaded* movement test set was longer so as to include the second trials with each of the four loads.

Once each ANN was trained using EMG, kinematics, and - for loaded movements - grip force data from the training set, EMG signals were then predicted using kinematics and, for loaded movements, grip force obtained during the test set as inputs to the trained ANN. The coefficient of determination (R^2^) based on the correlation between the actual and predicted EMG signals for each muscle during the test set was used to quantify the quality of the predictions made by each algorithm.

To better understand and to evaluate the importance of including external forces in the prediction of EMG signals, we also performed *cross* testing for which we predicted EMG signals for the test set of the *loaded* movements but based on training data obtained from the *unloaded* movements. Our expectation was that predictions in this case would be significantly degraded compared to that in which external forces were included in the training set. For completeness, we also did the inverse, namely, we predicted EMG signals from the test set of *unloaded* movements based on training data obtained from the *loaded* movements. Because the *loaded* movement training set included periods of zero load, our expectation was that prediction capability would be little affected in this case.

The primary statistical analysis was a repeated-measures analysis of variance (ANOVA) performed on R^2^ values using muscle and type of movement as factors. Post-hoc analyses using pair-wise multiple comparisons were executed using the Holm–Sidak method. Data are reported as means (±SD). The level chosen for significance in the statistical tests was p < 0.05.

## Results

**Figure 2:**
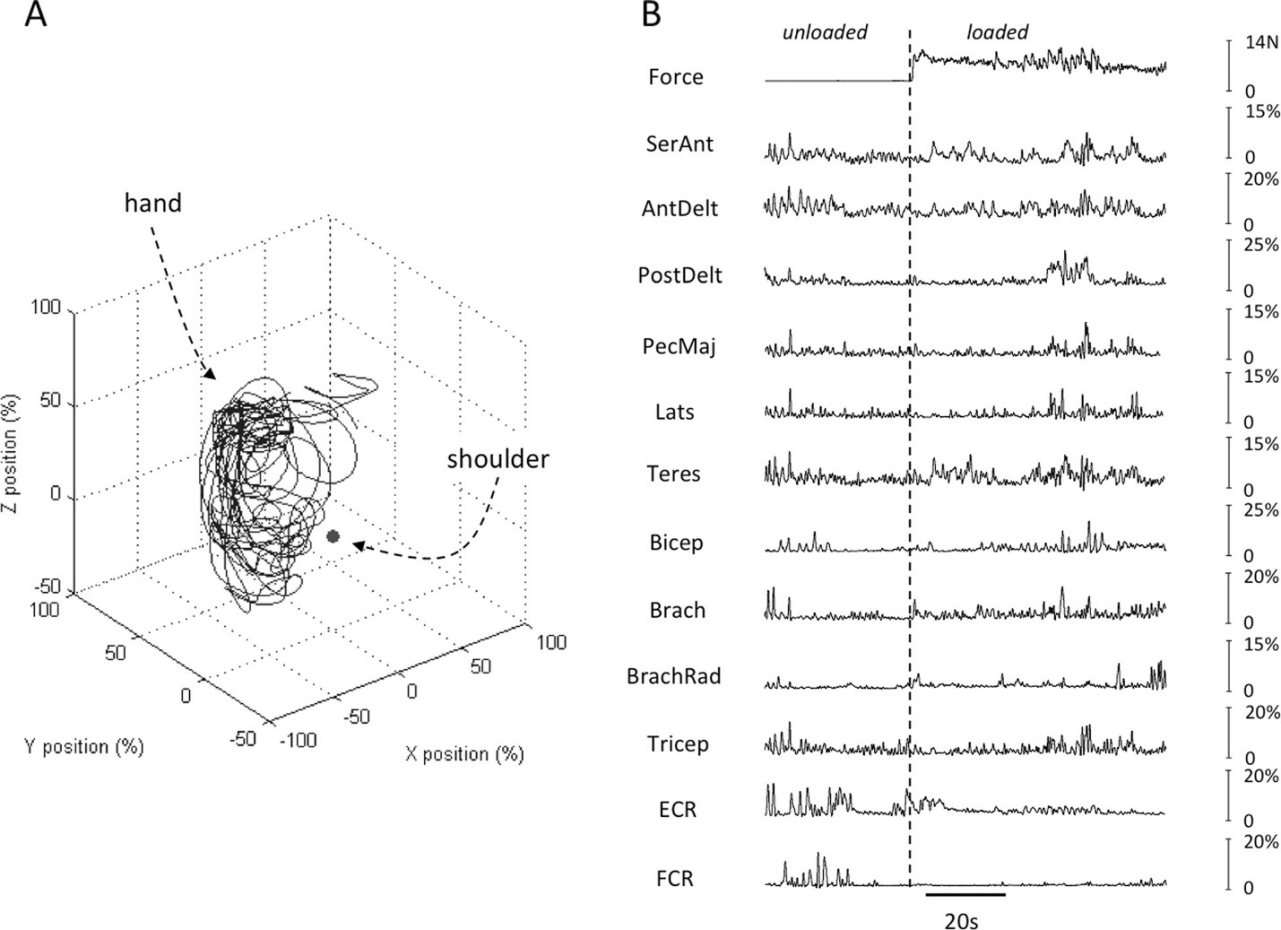
**Example segment (~100 s, loaded movements) of data recorded in one participant. (A)** Hand trajectory (X-axis: medial/lateral; Y-axis: anterior/posterior; Z-axis: superior/inferior relative to shoulder). **(B)** Grip force and rectified and smoothed EMG signals from 12 muscles (abbreviations as used in Figure 1). Vertical dashed line indicates time at which subject grasped the load (1000 g). Scale bars for EMG signals represent muscle activation normalized to that recorded during a maximum voluntary contraction for each muscle.

Figure 2 shows an example segment (~100 s) of the type of data used to train the ANNs associated with the *loaded* movement task. Figure 2A depicts the trajectory of the hand while Figure 2B shows the associated grip force and EMG signals recorded from the 12 muscles. About halfway through this example segment, the subject picked up a load (1000 g) and then continued to move the limb with the load in hand. Somewhat surprisingly, only a few muscles (e.g. elbow flexors) showed obvious changes in activity associated with lifting the load.

**Figure 3:**
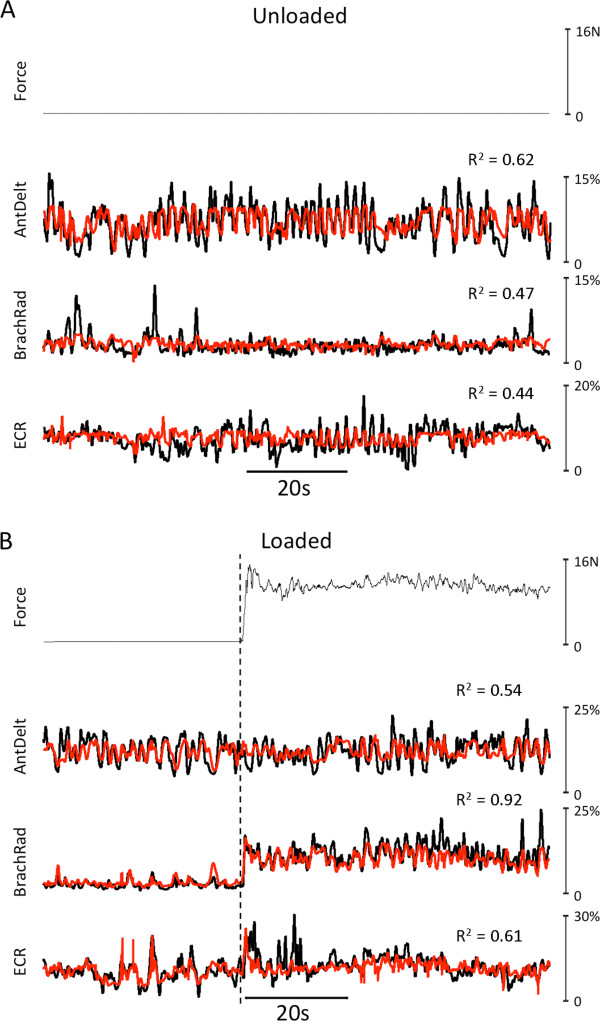
**Example predictions of EMG activity for representative shoulder, elbow, and wrist muscles.** Segment (~100 s) of EMG predictions (red traces) made by the MLP20_9_9_20 artificial neural network for anterior deltoid (AntDelt), brachioradialis (BrachRad), and extensor carpi radialis (ECR) for unloaded **(A)** and loaded movements **(B)**. Black traces indicate the actual (recorded) EMG signals. The test movement in B depicts an episode where the subject grasped and moved a load (1000 g, dashed vertical line). The coefficient of determination (R^2^) between predicted and actual EMG is shown for each muscle.

Example predictions of EMG signals for a shoulder (anterior deltoid), an elbow (brachioradialis), and a wrist muscle (extensor carpi radialis) generated by the most complex ANN (MLP20x9x9x20) are depicted in Figure 3. Figure 3A shows predictions for the *unloaded* movement task whereas Figure 3B shows predictions associated with the *loaded* movement task. In each muscle-activity panel, black traces indicate the actual EMG recorded during a representative 100 s segment of the test set whereas the red traces show the predicted signals computed based upon hand trajectory (Figure 3A) or hand trajectory and grip force (Figure 3B). Coefficient of determination values (R^2^) between actual and predicted EMG are indicated for each muscle. As shown previously [19], this ANN architecture predicted patterns of muscle activity reasonably well during *unloaded* movements of the limb (Figure 3A). Of the three example muscles depicted in Figure 3A, predictions were best for the shoulder muscle (anterior deltoid) and less so for the elbow and wrist muscles, similar to our previous findings [18, 19]. Also, as we have reported previously [18], the predicted levels of EMG tended to undershoot the actual values, particularly during transient, higher-magnitude activity.

Figure 3B shows example predictions of EMG signals for the same three muscles associated with the *loaded* movement task. This example segment includes a transition (vertical dashed line) from zero load to that associated with picking up and moving the 1000 g object. As for the unloaded movements, predictions of EMG were reasonable for these three muscles (R^2^ values > 0.4). Importantly, in these example muscles, there was no diminution in predictive capability of the ANN when the subject interacted with external loads (Figure 3B) compared to that associated with unloaded movements (Figure 3A). Indeed, for the elbow flexor brachioradialis (BrachRad), prediction appeared to improve for the loaded movements.

**Figure 4:**
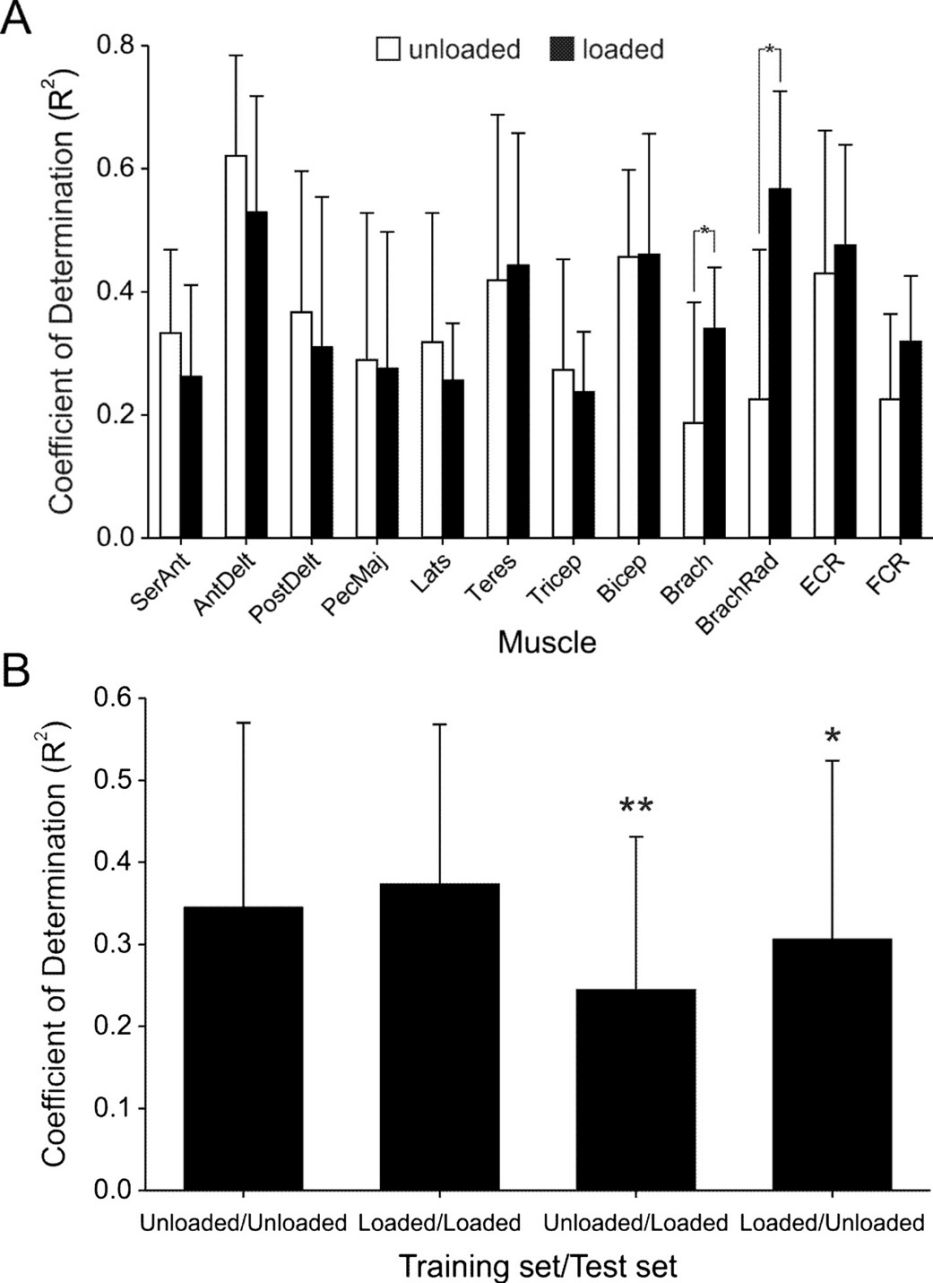
**Mean (SD) R** ^**2**^ **values between predicted and actual EMG signals. (A)** Mean R^2^ values across different muscles during unloaded (open bars) and loaded (filled bars) movements. ANOVA indicated no significant effect on R^2^ of movement type but a significant effect of muscle (P < 0.001) and a significant interaction (P < 0.001) between the factors muscle and movement type. Post hoc analysis indicated a significant (P < 0.01) difference (*) in R^2^ when comparing *loaded* to *unloaded* movements for just two muscles: brachialis and brachioradialis (muscle abbreviations as in Figure 1). **(B)** Effect of training set on predictions of different test data sets. Each bar represents mean (SD) R^2^ value across all subjects and muscles for each training set/test set combination. R^2^ for the condition in which the training set was obtained from *unloaded* movements and used to predicted *loaded*-movement EMG was significantly lower (P < 0.05, *) than that of all the other conditions. In addition, R^2^ values for the condition in which the training set was obtained from *loaded* movements but used to test *unloaded* movements were significantly lower (P < 0.05, **) than that when training and test sets both included included *loaded* movements (bar 2).

Figure 4A shows the mean (SD) R^2^ values for the nine subjects across all muscles and for the two movement types (*unloaded* - open bars, *loaded* - filled bars). Repeated measures two-way ANOVA revealed no significant effect of movement type (F = 0.90, P = 0.37) on R^2^. As such, the ability of the ANN to predict EMG was, in general, equally as good for tasks involving interacting with external loads as for unloaded movements. As we have seen before [18, 19], however, there was, a significant effect of muscle (F = 4.19, P < 0.001) on R^2^. Furthermore, there was a significant interaction (F = 6.54, P < 0.001) between the factors muscle and movement type such that prediction of EMG for a given muscle could depend on the type of movement performed. For *unloaded* movements, post hoc analysis indicated that the R^2^ value for the anterior deltoid (0.62 ± 0.16) was significantly (P < 0.001) greater than that of seven other muscles (serratus anterior, pectoralis major, latissimus dorsi, triceps, brachialis, brachioradialis, flexor carpi radialis). In addition, the R^2^ value for biceps (0.48 ± 0.15) was significantly (P < 0.001) greater than brachialis. No other pairwise comparisons were significant for the *unloaded* movements.

There was a tendency for higher R^2^ values in distal muscles when comparing *loaded* to *unloaded* movements (Figure 4A). This difference was significant, however, for just two muscles: brachialis (P = 0.007) and brachioradialis (P < 0.001). This tendency for improved prediction in distal muscles when subjects interacted with external loads presumably was due to the more direct influence that distally handled loads had on these muscles (e.g. see Figure 3B). Overall, the fit for brachioradialis (R^2^ = 0.57 ± 0.16) for *loaded* movements was significantly (P < 0.001) better than that of serratus anterior, pectoralis major, latissimus dorsi, and triceps. And, as was the case for unloaded movements, the R^2^ value for the anterior deltoid (0.53 ± 0.19) during the *loaded* movements was significantly greater (P < 0.001) than that of serratus anterior, latissimus dorsi, and triceps. The particularly high degree of correspondence between predicted and actual EMG signals for the anterior deltoid in these experiments and others [18, 19] is likely related to its critical role for generating torque at the shoulder during a wide range of upper limb movements [29].

To gain some insight as to the significance of including external force signals for predicting EMG, we predicted EMG signals for the test set of the *loaded* movements based on training data obtained from the *unloaded* movements. We also did the converse, namely, predicted EMG signals for the *unloaded* movement test set based on training data from the *loaded* movements. These results were then compared to those for which training and test sets were obtained from the same type of movements (as described above). Data for all muscles and subjects were combined for a given training/test set combination and evaluated using a one-way ANOVA.

Figure 4B shows the mean (SD) R^2^ values for these four conditions (i.e. training/test set combination). The ANOVA revealed a significant (P < 0.001) effect of training/test set combination on R^2^. Post hoc analysis indicated that R^2^ values for the condition in which the training set was obtained from *unloaded* movements and used to predict *loaded*-movement EMG was significantly lower (P < 0.05) than that of all the other conditions. This result indicates, as might be expected, that prediction of EMG signals associated with movements that include periods of object interaction are diminished if contact force measurements are not included in the training set. Post hoc analysis also indicated that R^2^ values based on *loaded*-movement training data were lower when the test set included only *unloaded* movements (bar 4, Figure 4B) compared to that when the test set included *loaded* movements (bar 2, Figure 4B).

**Figure 5:**
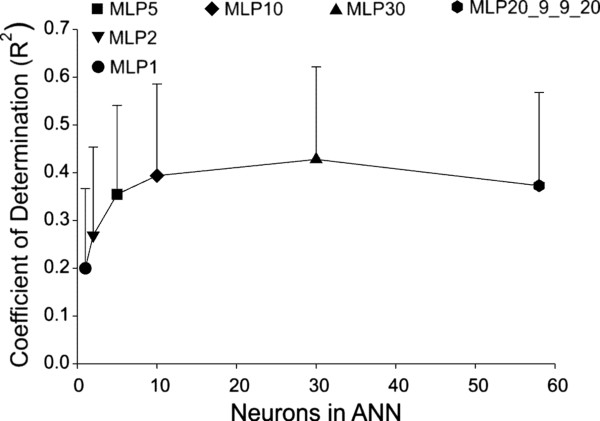
**Evaluation of different artificial neural network structures in ability to predict EMG.** Evaluations were performed using training and testing data obtained from loaded movements. Mean (SD) R^2^ values across all subjects and muscles using six artificial neural networks (MLP1, MLP2, MLP5, MLP10, MLP30, MLP20_9_9_20) and plotted as a function of the total number of neural elements in each network. The R^2^ value of the MLP30 network was significantly (P < 0.05) larger than that of every other network architecture tested.

A secondary goal of our study was to compare different ANNs that varied in complexity in their abilities to predict EMG signals. Thus, in addition to our original ANN with 4 hidden layers (MLP20_9_9_20, see Methods), we also tested ANNs with varying number of neurons in a single hidden layer (MLP1, MLP2, MLP5, MLP10, and MLP30). For each structure, we trained and tested using *loaded* movements only. R^2^ values for all muscles and subjects were combined for a given architecture and evaluated using a one-way ANOVA. This analysis indicated a significant (P < 0.001) effect of ANN structure on R^2^. Figure 5 shows the mean (SD) R^2^ values for each type of ANN plotted as a function of the total number of neurons included in the network. Surprisingly, the mean R^2^ value (0.37 ± 0.19) based on the most complex network (MLP20_9_9_20) was significantly (P < 0.05) less than that obtained with the simpler MLP30 network (R^2^ = 0.43 ± 0.19). Furthermore, the MLP20_9_9_20 network statistically performed no better than that achieved with the MLP10 or MLP5 networks. On the other hand, the predictive capability (i.e. R^2^) of the MLP30 network was significantly better than that of every other network architecture tested.

We also measured the average computer time required to train each network. The training times varied over an exceptionally wide range of values, from 5 minutes for the simplest network (MLP1) to 2718 minutes (i.e. 45.3 hours) for the most complex network (MLP20_9_9_20). The training time for the MLP30 network was 1860 min (31 hours); 68% of the time required for the MLP20_9_9_20 network. As such, the MLP30 network was computationally more efficient than the more complex network (MLP_20_9_9_20) and achieved better predictions of EMG than any of the ANNs tested. Consequently, the MLP30 network would seem to be the best overall choice to predict EMG from kinematics and contact force signals associated with a wide range of complex upper limb motor behaviors.

**Figure 6:**
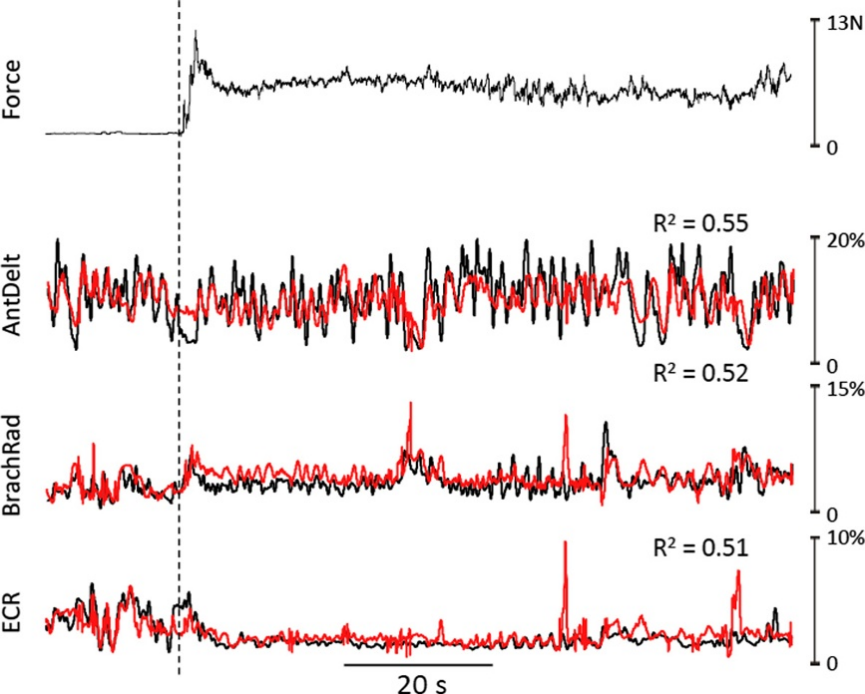
**Across-subject prediction of EMG activity.** Example segment (~100 s) of predicted EMG signals (red traces) made by the MLP30 artificial neural network for anterior deltoid (AntDelt), brachioradialis (BrachRad), and extensor carpi radialis (ECR). The network was trained using loaded movement data from one subject and was then used to predict muscle activity patterns in a different test subject. Top trace indicates the grip force and black traces in lower three panels indicate the actual (recorded) EMG signals in the test subject. The coefficient of determination (R^2^) between predicted and actual EMG is shown for each muscle.

To evaluate the degree to which this approach might be transferable across subjects, we trained the MLP30 network with data obtained from loaded movements of one subject and used it to predict EMG signals during loaded movements in a different test subject. Figure 6 shows predicted (red traces) activity levels in three representative muscles superimposed on the actual EMG signals (black traces) recorded in this test subject. While such across-subject predictions were not as good as those obtained within subjects (cf. Figure 3B), overall the predictions were still reasonable, with an average R^2^ value of 0.38 ± 0.12 across the 12 muscles.

## Discussion

Here we have shown, as has been demonstrated previously [30–32, 19], that EMG patterns associated with complex limb movements can be predicted with good fidelity from kinematics using artificial neural networks (ANNs). We have extended those findings here to show that the predictive capabilities of ANNs are retained for movements during which subjects grasp and move objects of varying weights and dimensions if grip force is included in the training of the ANN.

### Limitations

The development of a system for controlling functional electrical stimulation involves a balance between practical utility and accuracy of prediction. We have attempted to strike this balance partly by minimizing the number of signals needed to make reasonable predictions of muscle activity. In this and previous studies [18, 19], we have shown that hand position and orientation information alone are sufficient for making good predictions of activity patterns across several muscles during complex movements. Indeed, it has been surprising that inclusion of hand velocity and acceleration only modestly affected EMG-signal prediction [18]. Nevertheless, higher order kinematics might become important, for example, when moving a load. As such, it seems possible that even better predictions might have been achieved here had we included other kinematic parameters. This issue needs to be addressed in future investigations.

Likewise, the complex array of contact forces associated with grasping and manipulating objects was not captured in the present experiments. Instead, we recorded a single signal representing the normal forces applied to the thumb. While we argued (see Methods section) that the thumb normal force might provide a reasonable proxy for grip force and load magnitude, it certainly fails to capture important subtleties of individual digit forces and the directions of contact forces. Inclusion of contact forces (including tangent forces) from other digits might help improve prediction of EMG signals. Nevertheless, the present study provided a proof-of-concept that inclusion of contact-force information can improve prediction of muscle activity patterns.

It should be noted that the force sensor, while used here to provide an estimate of the external load applied to the hand, would also register inertial effects associated with limb acceleration. This effect can be seen in the force trace of Figure 2B where fluctuations in force (presumably due to inertial effects) are superimposed on the steady state level. In the context of an FES controller, such inertial forces (arising as an outcome of muscle stimulation) would be fed back to the control algorithm - giving rise to a closed loop that could cause some instability. The extent of this instability needs to be explored in future studies.

An important and additional potential limitation of this study has to do with the overall quality of the predictions. For loaded movements, the average R^2^ value across all muscles and subjects was 0.43 (using the MLP30 architecture). One must question whether this level of prediction accuracy (i.e. 43% of the variance in the actual EMG signals accounted for by the predicted EMG) would be sufficient to control an FES system. We are planning to test this question directly by comparing movement trajectories evoked with FES based on ANN-predicted patterns of EMG to that evoked using actual EMG signals. These experiments will help reveal the degree to which errors in EMG prediction are translated into inaccuracies in FES evoked-movements.

### Analytical vs. machine-learning prediction

Ostensibly, perhaps the most straightforward way to identify patterns of muscle activity associated with a wide range of movements would be to predict them using an analytical approach based on a biomechanical model of the limb. As such, one predicts the net torques generated at each joint for a given desired trajectory of the limb using inverse dynamics [33–35]. Net torques are resolved into individual muscle forces using anatomical models [36] and various optimization functions [37–40], which in turn, are transformed into estimates of activity levels [41–43] based on muscle mechanics and notions of excitation-contraction coupling [44–46]. In theory, these predicted patterns of muscle activity could then serve as templates for electrical stimulation needed to evoke the desired movements [47].

Blana et al. [48] used such an analytical approach to predict patterns of muscle activity associated with upper limb movements. Their sophisticated model included 29 muscles, 6 bones, 5 joints and host of physiological parameters. To evaluate the model, kinematics recorded from healthy subjects during simple arm movements were used as inputs to the model. EMG signals were also recorded from some arm muscles during the movements. These EMG signals were then compared to model-predicted patterns of muscle activity. Despite the comprehensiveness and rigor of this model, predictions of muscle activity were relatively poor.

Here we applied a machine-learning approach involving artificial neural networks as a non-analytical means to predict muscle activity associated with desired movements. Machine learning is an established field of computer science in which the characteristics of a system are learned based on data, rather than represented by programmed instructions. The practical advantage of machine learning is its simplicity. There is no need to specify explicit algorithms that represent the enormously complex set of interactions by which activities in several muscles are transformed into movement of a limb possessing multiple degrees of freedom. Furthermore, the predictions here were reasonable, with prediction accuracies better than that achieved by Blana et al. [48] using an analytical model.

### Network structure

A secondary objective of the present study was to evaluate the ability of different neural network architectures to predict EMG from kinematics and grip forces. Interestingly, the network structure that achieved the best overall predictions was not the most complex. Indeed, it possessed a single hidden layer comprised of 30 neural elements, whereas the most complex ANN tested consisted of almost double that number of neural elements distributed over four hidden layers. The most likely reason for the poorer performance of the more complex network was that it over-fit the specific data set used for training. As such, it likely began to predict noise in the training set rather than tracking the underlying relationships. Consequently, when tested on a new data set, predictions were partially degraded. In addition to the obvious advantage of better predictions with a simpler network, the computer time required to train simpler networks decreases logarithmically with fewer numbers of neural elements.

### Implications for neuroprosthetics

The long-term goal of this work is to develop an upper limb neuroprosthetic to greatly expand motor function in individuals paralyzed as a consequence of spinal cord injury or stroke. As we have shown here and elsewhere [17–19], machine-learning methods can be used to predict complex patterns of muscle activity associated with a wide array of upper-limb movements based primarily on hand trajectory information. In the present study, we have also shown that inclusion of tactile force signals enables accurate estimation of EMG signals during grasping and movement of objects with the hand. It seems feasible, therefore, that predicted patterns of muscle activity associated with a desired limb trajectory and hand contact forces could be translated into patterns of amplitude and frequency modulated stimulus pulses [20] to elicit movements in paralyzed subjects. Desired movement trajectories (which serve as inputs to the ANN) could be identified in a number of ways. For example, EMG activity recorded from non-paralyzed muscles that are naturally engaged during attempted movements can be used to predict desired limb kinematics [49, 50]. Also, in the case of stroke-related hemiplegia, desired movements of the paretic limb could be detected from sensors placed on the contralateral unimpaired limb [51]. It also seems feasible that straight-line reach trajectories to targets in peri-personal space could be identified using glasses-mounted displays and eye-tracking devices. Ultimately, desired hand trajectories could also be identified directly from recordings made in the cerebral cortex [52–56]. Such cortical control of FES could further capitalize on learning and neural plasticity to partially adjust for errors in the transformation of desired movements into patterns of muscle stimulation. If combined with contact forces detected with tactile sensors, such an integrated system could provide appropriate patterns of muscle stimulation needed to elicit desired motor behaviors and thereby reinstate a measure of voluntary control over the patient’s own limb.

## Conclusion

The results of this study indicate that ANNs could be implemented as a simple means to predict complex patterns of muscle stimulation needed to produce a wide array of movements, including those involving object interaction, in paralyzed individuals using functional electrical stimulation.
